# The therapeutic utility of combining dynamic contrast-enhanced magnetic resonance imaging with arterial spin labeling in the staging of nasopharyngeal carcinoma

**DOI:** 10.1186/s12880-023-01016-3

**Published:** 2023-05-03

**Authors:** Haodong Li, Guanzhong Gong, Lizhen Wang, Ya Su, Jie Lu, Yong Yin

**Affiliations:** 1 Department of Graduate, Shandong First Medical University (Shandong Academy of Medical Sciences), Jinan, 250000 China; 2 Department of Radiation Physics, Shandong Cancer Hospital and Institute, Shandong First Medical University and Shandong Academy of Medical Sciences, Jinan, 250117 China

**Keywords:** DCE-MRI, Arterial spin labeling, Nasopharyngeal carcinoma, Clinical staging

## Abstract

**Background:**

To research the pathological and clinical staging uses of arterial spin labeling (ASL) and dynamic contrast-enhanced magnetic resonance imaging (DCE-MRI).

**Materials and methods:**

64 newly diagnosed nasopharyngeal carcinoma (NPC) patients were enrolled from December 2020 to January 2022, and 3.0 T MRI (Discovery 750W, GE Healthcare, USA) were used for ASL and DCE-MRI scans. The DCE-MRI and ASL raw data were processed post-acquisition on the GE image processing workstation (GE Healthcare, ADW 4.7, USA). The volume transfer constant (Ktrans), blood flow (BF), and accompanying pseudo-color images were generated automatically. Draw the region of interest (ROIs), and the Ktrans and BF values for each ROI were recorded separately. Based on pathological information and the most recent AJCC staging criteria, patients were divided into low T stage groups = T_1–2_ and high T stage groups = T_3–4_, low N stage groups = N_0–1_ and high N stage groups = N_2–3_, and low AJCC stage group = stage I–II and high AJCC stage group = stage III–IV. The association between the Ktrans_t_ and BF parameters and the T, N, and AJCC stages was compared using an independent sample t-test. Using a receiver operating characteristic (ROC) curve, the sensitivity, specificity, and AUC of Ktrans_t_, BF_t_, and their combined use in T and AJCC staging of NPC were investigated and assessed.

**Result:**

The tumor-BF (BF_t_) (t = − 4.905, P < 0.001) and tumor-Ktrans (Ktrans_t_) (t = − 3.113, P = 0.003) in the high T stage group were significantly higher than those in the low T stage group. The Ktrans_t_ in the high N stage group was significantly higher than that in the low N stage group (t = − 2.071, P = 0.042). The BF_t_ (t = − 3.949, P < 0.001) and Ktrans_t_ (t = − 4.467, P < 0.001) in the high AJCC stage group were significantly higher than those in the low AJCC stage group. BF_t_ was moderately positively correlated with the T stage (r = 0.529, P < 0.001) and AJCC stage (r = 0.445, P < 0.001). Ktrans_t_ was moderately positively correlated with T staging (r = 0.368), N staging (r = 0.254), and AJCC staging (r = 0.411). There was also a positive correlation between BF and Ktrans in gross tumor volume (GTV) (r = 0.540, P < 0.001), parotid (r = 0.323, P < 0.009) and lateral pterygoid muscle (r = 0.445, P < 0.001). The sensitivity of the combined application of Ktrans_t_ and BF_t_ for AJCC staging increased from 76.5 and 78.4 to 86.3%, and the AUC value increased from 0.795 and 0.819 to 0.843, respectively.

**Conclusion:**

Combining Ktrans and BF measures may make it possible to identify the clinical stages in NPC patients.

## Background

Nasopharyngeal carcinoma (NPC) has the highest rate of tumors of the head and neck in Southeast Asia [1, 2]. Radiotherapy is the main treatment for NPC [3, 4]. For the individualization of treatment and the forecasting of patient outcomes, accurate clinical staging before therapy is essential. Since MRI has the highest resolution of soft tissue and the highest sensitivity for NPC identification, it has been the preferred imaging modality for NPC staging [5]. Unfortunately, morphology-based imaging methods cannot detect tumor microbiological information. As a result, it might compromise the precision of NPC staging.

Traditional anatomy-based imaging methods may have evaluation errors [6], therefore the MR functional pictures based on dynamic contrast-enhanced magnetic resonance imaging (DCE-MRI) and arterial spin labeling (ASL) are crucial for determining the effectiveness of treatment for NPC, staging accurately, and other purposes [7].

Exogenous Gd-contrast agents are required for DCE-MRI. It accumulates in the extracellular space, which is dictated by tissue perfusion, capillary permeability, and surface area, and it may perform visual or semi-quantitative analysis [8, 9]. DCE-MRI can collect a variety of information on tissue perfusion, including the ability to objectively measure capillary permeability, NPC perfusion, tumor hypoxia, and tumor biological activity using temporal signal intensity curves [10]. When compared to the role of MR anatomical images in the diagnosis of NPC, DCE-MRI can further improve the capacity of diagnosis, differential diagnosis, and precise staging.

Unlike DCE-MRI perfusion, ASL is fully non-invasive and does not require an injection of a contrast agent. Using magnetically tagged water protons from arterial blood as an endogenous tracer, this method creates images that can show microscopic modifications such as tissue blood perfusion and micro-vessel density [11]. ASL’s primary physiological parameter is blood flow (BF), which determines the rate at which oxygen and nutrients are transported to the capillary bed and provides physiological information on tissue blood perfusion [12].

These two techniques have been utilized to stage NPC and predict therapy response or prognosis [5, 7, 13]. As a result, the combination of ASL and DCE-MRI is promising for reflecting the type of NPC and has the potential to improve staging accuracy. In this study, the two imaging techniques DCE-MRI and ASL were used to stage NPC to investigate the function of DCE-MRI and ASL in determining the staging of NPC.

## Materials and methods

The Shandong Cancer Hospital Medical Ethics Committee approved the study (NO. SDTHEC2022006002). Before the MR examination, all patients provided informed consent and signed. 77 newly diagnosed NPC patients underwent MRI scans between December 2020 and January 2022. Due to the image quality of conventional sequences will be affected by noise, motion artifacts, and metal foreign body artifacts created by patients during MR testing, there were no artifacts in either the ASL or DCE-MRI scans and 13 patients were excluded. Finally, 64 patients were included. All participants in this study met the requirements listed below:

- Biopsy-proven diagnosis of NPC;
- No history of prior chemotherapy, radiotherapy, or other treatments;
- No contraindications for MR examination;
- No concurrent nasopharyngeal disease or other tumors.

Exclusion criteria:

- patients with recurrent NPC after treatment;
- MR image quality is poor (noise, motion artifacts, and metallic foreign body artifacts are the main causes), as long as there are artifacts visible in each sequence image, the image does not meet the requirements.

The pathology diagnosis for all individuals was NPC which was either poorly differentiated or undifferentiated. There were 64 patients (45 males and 19 women; mean age, 51 years; range, 14–80 years). The American Joint Committee on Cancer (AJCC) staging approach was used for the tumor, node, and metastasis TNM staging. Together with their clinical demographic information, the distribution of each patient's individual AJCC and TNM stages are listed in Table 1.

**Table 1:** Clinical characteristics of 64 patients

Parameters	Results (%)
Sex(male/female)	45(70%)/19(30%)
Age(years)	51
Range(years)	14–80
T stage	
T1	9(14%)
T2	23(36%)
T3	22(34%)
T4	10(16%)
N stage	
N0	9(14%)
N1	16(25%)
N2	17(27%)
N3	22(34%)
M stage	
M0	63(98%)
M1	1(2%)
AJCC stage	
I	0(0%)
II	13(20%)
III	23(36%)
IV	28(44%)

*AJCC* American Joint Committee on Cancer

## MR imaging protocol

The 3.0 T MRI (Discovery 750W, GE Healthcare, USA) Scanner was used for all of the patients’ MRI scans. Supine MR scanning specifications: all patients were scanned from the clavicle to the superior edge of the frontal sinus. Six-channel head and neck coils were used. The imaging protocol included the following: (1) axial T1 weighted images (fast spin echo, FSE): repetition time/echo time (TR/ TE) = 8.5/3.2 ms, thickness = 3 mm, slice gap = 1 mm, field of view (FOV) = 25.6 cm, matrix = 256 × 256; (2) axial T2-weighted images (fast spin echo, FSE): TR/TE = 13,500/114 ms, thickness = 3 mm, slice gap = 1 mm, FOV = 26 cm, matrix = 416 × 416; (3) axial T2 with fat suppression (fast recovery fast spin echo, FRFSE): TR/TE = 11,000/114 ms, thickness = 3 mm, slice gap = 1 mm, FOV = 28 cm, matrix = 320 × 320; (4) axial ASL (3D fast spin echo spiral-based pseudo-continuous ASL sequence): TR/TE = 5160/11.5 ms, NEX = 3, bandwidth = 62.5 kHz, thickness = 3 mm, FOV = 25.6 cm, labeling duration = 1500 ms, post-labeling delay = 2025 ms. (5) axial DCE-MRI imaging (gradient echo sequence, GRE): TR/TE = 5.5/2.7 ms, thickness = 3 mm, slice gap = 0 mm, FOV = 40 × 32 cm, matrix = 300 × 180. An intravenous injection of 0.2 mmol/kg gadolinium acid meglumine salt (Jiangsu Hengrui Pharmaceuticals Co., Ltd. Batch number: 220519BT) was carried out at an injection rate of 3 ml/s via a power injector, followed by a flush of an equal volume of normal saline. Another ASL was acquired after the completion of DCE-MRI.

## Postprocessing and evaluation

**Fig. 1:**
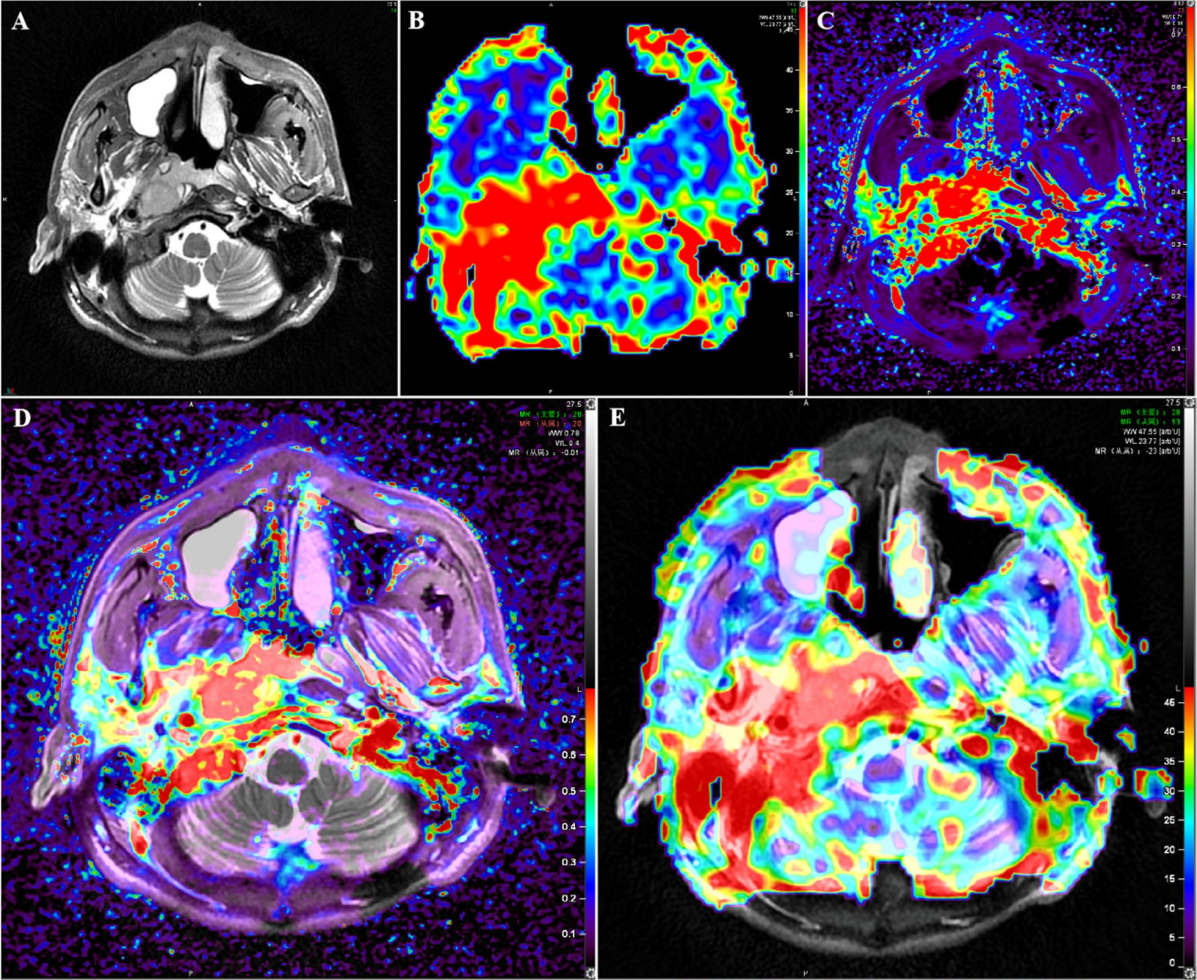
Schematic diagram of MRI of a patient. **A** T2WI-FS; **B** ASL(BF); **C** DCE-MRI(Ktrans); **D** fusion images of T2WI-FS and ASL(BF); **E** fusion images of T2WI-FS and DCE-MRI(Ktrans)

Two senior radiologists used MIM Maestro 6.8.8 to delineate the parotid gland, lateral pterygoid muscle, and gross tumor volume (GTV) in T2WI-FS images, which was then reviewed by a third senior radiologist. The GE image processing workstation (GE Healthcare, ADW 4.7, USA) performs post-processing on the DCE and ASL raw data (Fig. 1). Pseudo-colored maps with the matching Ktrans and BF parameters were created automatically after being computed separately (Fig. 1B, C). Because Ktrans is the most typical metric in DCE, we solely measured it. The BF and Ktrans maps were rigidly registered to the axial T2WI-FS at a workstation using commercial software (GE Healthcare, ADW 4.7, USA) (Fig. 1D, E). The regions of interest (ROIs) were manually drawn by one radiologist with > 10 years of clinical experience in head and neck radiology. According to the pseudo-color perfusion of images, the ROIs were set at the slice with the highest perfusion signal while avoiding areas of cysts, necrosis, calcifications, hemorrhage, and large vessels. Three separate ROIs were obtained at the largest cross-section of the lesion, and the average value was taken as the final measurement result. Each ROI’s BF and Ktrans values were recorded.

A sequence with pseudo-continuous labeling, background suppression, and 3D RARE Stack-Of-Spirals readout was implemented for this study. Obtaining ASL perfusion pictures and measuring arterial blood magnetization. To eliminate physiological noise and motion artifacts, background suppression technology was applied during the image collecting procedure. After scanning, the quantitative BF maps were created automatically using the GE FuncTool 4.7 software using the following equation:

$$\mathrm{BF}=\frac{6000\uplambda \left({\mathrm{M}}_{\mathrm{c}}-{\mathrm{M}}_{\mathrm{l}}\right)\frac{\mathrm{PLD}}{{\mathrm{e}}^{{\mathrm{T}}_{1\mathrm{Blood}}}}}{2\mathrm{\alpha }{\mathrm{T}}_{1\mathrm{Blood}}{\mathrm{M}}_{\mathrm{PD}}\left(1-{\mathrm{e}}^{\left(\frac{\uptau }{{\mathrm{T}}_{1\mathrm{Blood}}}\right)}\right)}$$

where λ is the nasopharynx/blood partition coefficient in ml/g and M_c_ and M_l_ are the time-averaged signal intensities in the control and label images respectively. T_1Blood_ is the longitudinal relaxation time of blood in seconds, α is the labeling efficiency, M_PD_ is the signal intensity of a proton density-weighted image, $\uptau$ is the label duration, and PLD is the post-labeling delay.

DCE-MRI data can be analyzed using Tofts standard pharmacokinetic model, which gives the paramagnetic contrast agent (CA) concentration in tissue at time t as follows:

$${\mathrm{C}}_{\mathrm{t}}\left(\mathrm{t}\right)={\mathrm{K}}_{\mathrm{trans}}{\int }_{0}^{\mathrm{t}}{\mathrm{C}}_{\mathrm{p}}\left(\uptau \right){\mathrm{e}}^{-\left(\frac{{\mathrm{K}}_{\mathrm{trans}}}{{\mathrm{V}}_{\mathrm{e}}}\right)\left(\mathrm{t}-\uptau \right)}\mathrm{d\tau }$$

where V_e_ is the extravascular extracellular volume percentage, Ktrans is the transfer rate constant, and C_t_(t) is the CA concentration in arterial blood.

The patients were classified into low T stage group = T_1-2_, high T stage group = T_3-4_, low N stage group = N_0-1_, high N stage group = N_2-3_, low AJCC stage group = stage I-II, and high AJCC stage group = stage III-IV based on the pathological findings and the most recent AJCC staging criteria.

## Statistical analysis

The data were examined using SPSS (version 25.0, IBM, NY, USA), a statistical tool. Measurement data with a normal distribution are represented by the mean ± standard deviation. The Ktrans and BF parameters of the T, N, and AJCC stages were compared using an independent sample t-test. The relationship between MRI parameters and the clinical stage was investigated using Spearman correlation analysis. To identify and assess the sensitivity, specificity, and AUC of Ktrans_t_, BF_t_, and their combination in T, N, and AJCC staging of NPC, the receiver operating characteristic curve (ROC) was used.

## Results

In total, 64 people with NPC were included in this study. 32 patients were in the low T stage (T_1–2_), and 32 people were in the high T stage (T_3–4_). There were 25 (39%) and 39 (61%) people with low N stage (N0-1) and high N stage, respectively (N_2–3_). Low AJCC stage (I-II stage) patients made up 13 (20%), while high AJCC stage (III–IV stage) patients made up 51 (80%) (Table 1).

The characteristics of the low-stage and high-stage groups are compared for DCE and ASL (Table 2). In comparison to the low T stage group, BF_t_ (t =  − 4.905, P = 0.001) and Ktrans_t_ (t =  − 3.113, P = 0.003) were significantly higher in the high T stage group. Ktrans_t_ was noticeably higher in the group with high N stages than in the group with low N stages (t = -2.071, P = 0.042). In comparison to the low AJCC stage group, BF_t_ (t =  − 3.949, P = 0.001) and Ktrans_t_ (t =  − 4.467, P = 0.001) were considerably greater in the high AJCC stage group. Between the T, N, and AJCC staging groups, there was no statistically significant difference in parotid gland-BF (BF_p_), parotid gland-Ktrans (Ktrans_p_), lateral pterygoid muscle-BF (BF_l_), or lateral pterygoid muscle-Ktrans (Ktrans_l_). BF_t_ did not significantly differ in the N stage group.

**Table 2:** Comparison of BF and Ktrans values across different clinical stages

Staging classification	Cases	BF_t_	Ktrans_t_	BF_p_	Ktrans_p_	BF_l_	Ktrans_l_
T stage							
T1 + T2	32	47.15 ± 17.94	0.79 ± 0.43	17.80 ± 5.59	0.69 ± 0.65	15.36 ± 2.80	0.21 ± 0.15
T3 + T4	32	67.56 ± 14.58	1.24 ± 0.69	15.71 ± 2.90	0.81 ± 0.58	14.81 ± 1.87	0.19 ± 0.15
*t*		− 4.905	− 3.113	1.873	− 0.789	0.926	0.495
*P*		0.000*	0.003*	0.067	0.433	0.358	0.648
N stage							
N0 + N1	25	52.92 ± 21.17	0.82 ± 0.50	17.18 ± 5.01	0.82 ± 0.70	15.23 ± 2.67	0.21 ± 0.19
N2 + N3	39	60.49 ± 17.30	1.14 ± 0.65	16.48 ± 4.26	0.70 ± 0.56	14.99 ± 2.22	0.20 ± 0.11
*t*		− 1.565	− 2.071	0.601	0.759	0.388	0.371
*p*		0.123	0.042*	0.550	0.451	0.700	0.712
AJCC stage							
I + II	13	40.70 ± 14.49	0.60 ± 0.37	18.40 ± 5.89	0.82 ± 0.87	15.64 ± 3.03	0.21 ± 0.18
III + IV	51	61.82 ± 17.81	1.12 ± 0.62	16.34 ± 4.09	0.73 ± 0.54	14.94 ± 2.21	0.20 ± 0.14
*t*		− 3.949	− 4.467	1.476	0.099	0.940	0.099
*p*		0.000*	0.000*	0.145	0.921	0.351	0.921

*BF*_*t*_ tumor-BF; *Ktrans*_*t*_ tumor-Ktrans; *BF*_*p*_ parotid gland-BF; *Ktrans*_*p*_ parotid gland-Ktrans; *BF*_*l*_ lateral pterygoid muscle-BF; *Ktrans*_*l*_ lateral pterygoid muscle-Ktrans

*P < 0.05

**Fig. 2:**
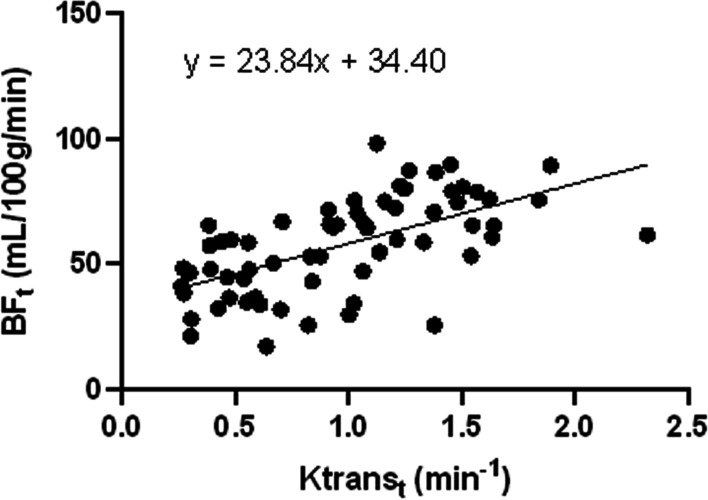
Scatterplot of BF_t_ and Ktrans_t_. There was a moderate positive correlation between BF and Ktrans in the tumor area

**Fig. 3:**
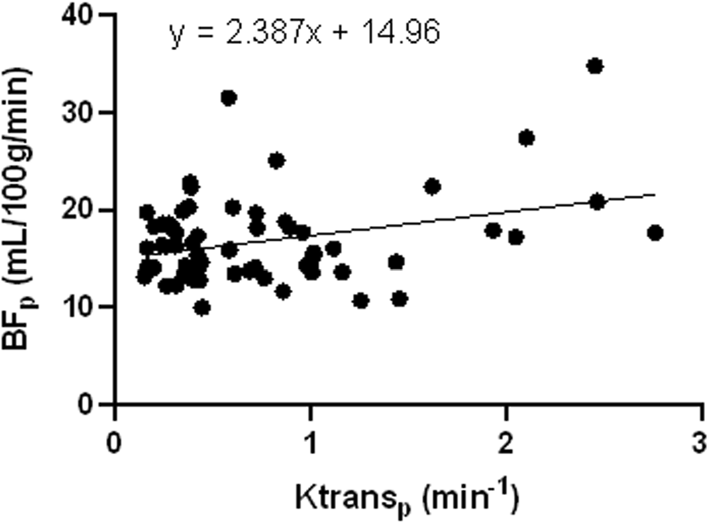
Scatterplot of BF_p_ and Ktrans_p_. There was a moderate positive correlation between BF and Ktrans in the parotid gland

**Fig. 4:**
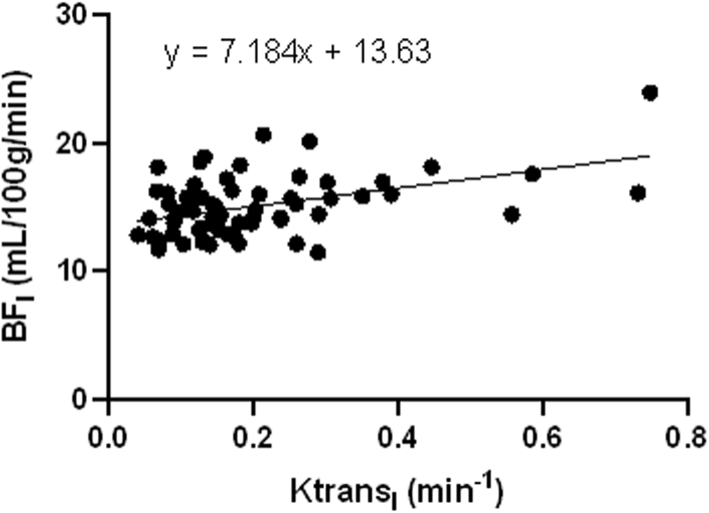
Scatterplot of BF_l_ and Ktrans_l_. There was a moderate positive correlation between BF and Ktrans in the Lateral pterygoid muscle

Tables 3 and 4 depicts the association between MR parameters and stage. BF_t_ value was associated with the T stage (r = 0.529, P < 0.001) and AJCC stage (r = 0.445, P < 0.001). The T stage (r = 0.368, P = 0.003), N stage (r = 0.254, P = 0.042), and AJCC stage (r = 0.411, P = 0.001) were all positively correlated with the Ktrans value. Ktrans and BF had a positively correlated with the GTV (r = 0.540, P < 0.001), parotid gland (r = 0.323, P = 0.009), and lateral pterygoid muscle (r = 0.445, P < 0.001) (Figs. 2, 3, and 4). There was no link found between BF_p_, Ktrans_p_, BF_l_, and Ktrans_l_ and AJCC stage.

**Table 3:** Correlation of BF and Ktrans values with stage

Parameters	T stage	N stage	AJCC stage
BF_t_			
*r*	0.529	0.206	0.445
*P*	< 0.001*	0.102	< 0.001*
Ktrans_t_			
*r*	0.368	0.254	0.411
*P*	0.003*	0.042*	0.001*
BF_p_			
*r*	− 0.140	− 0.077	− 0.159
*P*	0.268	0.545	0.210
Ktrans_p_			
*r*	0.147	− 0.029	− 0.096
*P*	0.246	0.817	0.452
BF_l_			
*r*	− 0.045	− 0.015	− 0.062
*P*	0.725	0.908	0.626
Ktrans_l_			
*r*	− 0.107	0.088	0.026
*P*	0.402	0.492	0.837

*BF*_*t*_ tumor-BF; *Ktrans*_*t*_ tumor-Ktrans; *BF*_*p*_ parotid gland-BF; *Ktrans*_*p*_ parotid gland-Ktrans; *BF*_*l*_ lateral pterygoid muscle-BF; *Ktrans*_*l*_ lateral pterygoid muscle-Ktrans

*P < 0.05

**Table 4:** Correlation between the BF values of the tumor, parotid gland, and lateral pterygoid muscle and their Ktrans

	BF_t_-Ktrans_t_	BF_p_-Ktrans_p_	BF_l_-Ktrans_l_
*r*	0.540	0.323	0.445
*P*	< 0.001*	0.009*	< 0.001*

*BF*_*t*_ tumor-BF; *Ktrans*_*t*_ tumor-Ktrans; *BF*_*p*_ parotid gland-BF; *Ktrans*_*p*_ parotid gland-Ktrans; *BF*_*l*_ lateral pterygoid muscle-BF; *Ktrans*_*l*_ lateral pterygoid muscle-Ktrans

*P < 0.05

**Fig. 5:**
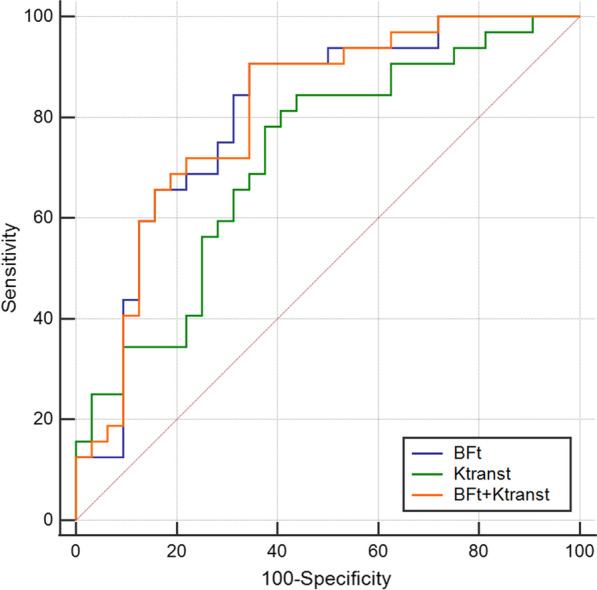
ROC curve analysis for T staging of NPC using the BF_t_, Ktrans_t,_ and both in combination. The AUCs of the BF_t_, Ktrans_t_, and BF_t_ + Ktrans_t_ were 0.806, 0.719, and 0.808, respectively. BF_t_ + Ktrans_t_ achieved the highest AUC

**Fig. 6:**
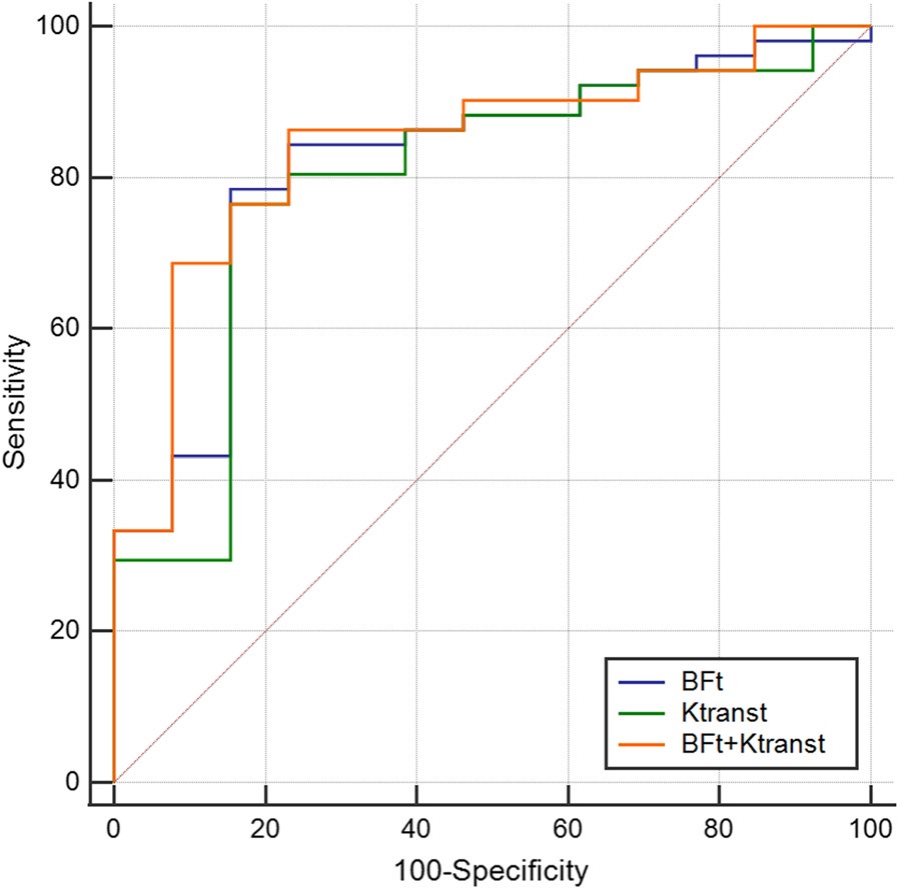
ROC curve analysis for AJCC staging of NPC using the BF_t_, Ktrans_t,_ and both in combination. The AUCs of the BF_t_, Ktrans_t_, and BF_t_ + Ktrans_t_ were 0.819, 0.795, and 0.843, respectively. BF_t_ + Ktrans_t_ achieved the highest AUC

Table 5 displays the sensitivity, specificity, and AUC of T staging and AJCC staging when Ktrans and BF are used alone and in combination. The AUC value was 0.806 when 50.38 mL/100 g/min was utilized as the cutoff value for the BF in the differentiation between the low and high T stages. The AUC value was 0.719 when 0.698 min^−1^ was utilized as the cutoff value for the Ktrans in the differentiation between the low and high T stage. The combination of Ktrans and BF had AUC values of 0.808. There was no statistically significant difference in AUC values (P > 0.05). The AUC value was 0.819 when 48.10 mL/100 g/min was utilized as the cutoff value for BF in the distinction of low and high AJCC stage. The AUC value was 0.795 when 0.698 min^−1^ was utilized as the cutoff value for the Ktrans in the distinction of low and high AJCC stage. The combination of Ktrans and BF had AUC values of 0.843. There was no statistically significant difference in AUC values (P > 0.05) (Figs. 5 and 6).

**Table 5:** Diagnostic sensitivity and specificity of NPC T and AJCC staging using BF_t_ and Ktrans_t_ alone or in combination

Parameters	T stage			AJCC stage		
	Sensitivity (%)	Specificity (%)	AUC	Sensitivity (%)	Specificity (%)	AUC
BF_t_	90.6	65.6	0.806	78.4	84.6	0.819
Ktrans_t_	84.4	56.3	0.719	76.5	84.6	0.795
BF_t_ + Ktrans_t_	90.6	65.6	0.808	86.3	76.9	0.843

*NPC* nasopharyngeal carcinoma; *AJCC* American Joint Committee on Cancer; *Ktrans*_*t*_ volume transfer constant; *BF*_*t*_ tumor blood flow

## Discussion

ASL and DCE were employed alone or in combination to assess the clinical stage of NPC in this investigation. BF and Ktrans were found to be useful in distinguishing between high and low T stages and high and low AJCC stages, with a substantial connection. The combination of ASL and DCE is more effective than either alone for the accurate staging of NPC.

By using data on blood perfusion and capillary permeability, DCE-MRI and ASL can both assist in determining the stage of NPC. According to recent studies, high T stage and high AJCC stage tumors showed higher BF values than low T stage and low AJCC stage cancers [14]. Sriyook et al.’s [15] research revealed a significant difference in Ktrans between the low T and high T stage. We found that the BF_t_ and Ktrans_t_ of the high T stage were higher than those of the low T stage. With increasing T stage, the tumor region's capillary permeability and blood perfusion increased, and the high T stage tumor cells were more active than the low T stage tumor cells. There was no discernible variation in the BF_t_ value even though the Ktrans_t_ of the high N stage was slightly higher than that of the low N stage. We believe that this parameter has no bearing on the lymph node metastasis of NPC and that the metastasis may only be marginally impacted by the blood supply of the tumor site.

It was recently discovered that the mean values of Ktrans, rate constant (K_ep_), and blood plasma volume (V_p_) in the III-IV stages were much greater than those in the I-II stages. Ktrans, K_ep_, and V_p_ were found to be strongly linked with tumor stage using univariate logistic regression analysis [16]. Our research came to a similar conclusion. The Ktrans_t_ and BF_t_ of the high AJCC stage were significantly greater than those of the low AJCC stage. The high-stage tumors differ from low-stage tumors in that they spread more quickly, grow more invasively, and require larger blood flow to provide nourishment and oxygen to the tumor location. DCE-MRI and ASL can therefore predict the clinical stage of NPC by assessing BF and Ktrans. The Ktrans and BF values for the lateral pterygoid muscle and parotid gland in each stage group did not significantly change. Because the majority of the patients we recruited were at stage M0, the tumor did not invade the parotid gland or the lateral pterygoid muscle. As a result, tumor size, depth of invasion, and lymph node metastasis may not have a substantial effect on BF and Ktrans that OARs.

According to some studies, a strong correlation between ASL and DCE-MRI in the perfusion of NPC [17]. According to Zheng et al., Ktrans had a moderately negative correlation with UICC staging, T staging, and N staging (r = − 0.447, r = − 0.244, and r = − 0.247, respectively) [18]. Researchers Sriyook Aniwat et al. found a correlation between plasma EBV-DNA levels and NPC stages and DCE-MRI features. Ktrans and T stage had the greatest correlation coefficient. The high T stage (T3-T4), low T stage (T1–T2), low AJCC stage (I–II stage), and high AJCC stage (III-IV stage) may all be distinguished with accuracy by Ktrans [15]. In this study, it was also found that, with the exception of the N stage, T and AJCC stages were moderately positively linked with BF_t_, whereas T, N, and AJCC stages were moderately positively linked with Ktrans_t_. In the parotid gland, lateral pterygoid muscle, and tumor region, Ktrans and BF were correlated. While BF mainly reflected blood perfusion, Ktrans mainly indicated increased new capillary permeability, which led to higher blood perfusion. The high stage tumors were more permeable to neovascularization, their tumor foci had an ample blood supply, and more contrast media accumulated. In comparison to tumor tissue, there was also a correlation between BF and Ktrans in normal tissue (parotid gland and lateral pterygoid muscle), which implied that the higher the capillary permeability was in the place of high blood perfusion in normal tissue. However, the tumor did not infiltrate the parotid gland or the lateral pterygoid muscle, and blood perfusion and capillary permeability were normal, thus the majority of them were in low BF and low Ktrans locations (Figs. 2, 3, and 4). Combining DCE-MRI with ASL analysis may help in the study of vascular permeability and blood perfusion in NPC. It can be applied as an extra measurement to evaluate tumor blood flow, stage the tumor, and provide a qualitative NPC diagnosis.

When used together, ASL and DCE-MRI can improve the diagnostic accuracy of MRI in the grading and staging of brain tumors, parotid tumor subtype distinction, and NPC [19–21]. The results of this study’s ROC analysis showed that combining Ktrans and BF produced staging results for T and AJCC that were more precise than either method used alone. The AJCC staging’s AUC outperformed T staging by a wide margin. Combination detection has a significantly higher sensitivity than solo detection for AJCC staging. The stage of the tumor may be more accurately determined if Ktrans and BF are detected simultaneously.

This research has some limitations. To begin with, the sample size is small. Because of the small sample size and the small number of people in the M1 stage, there is no statistical analysis of the M stage. In the future, we plan to collect more data on patients with NPC, particularly those with distant metastasis, to undertake additional studies. Second, Ktrans is the most commonly utilized in clinical settings and is the most representative of DCE [22]. Therefore, our study only looks at how the Ktrans parameters and BF relate to one another. In upcoming research, the relationship between ASL (BF) and other DCE parameters (V_e_, K_ep_, V_p_, etc.) will be further discussed.

## Conclusions

In conclusion, Ktrans values acquired from DCE-MRI combined with BF values derived from ASL might be utilized to assess capillary permeability and perfusion of NPC and offer helpful data for assessing stages in NPC patients.

## Data Availability

The datasets used and analyzed during this study are available from the corresponding author upon reasonable request. The data are not publicly available due to information that could compromise the privacy of research participants.

## Abbreviations

ASL: Arterial spin labeling
DCE-MRI: Dynamic-contrast-enhanced magnetic resonance imaging
MRI: Magnetic resonance imaging
Ktrans: Volume transfer constant
K_ep_: Rate constant
V_p_: Blood plasma volume
NPC: Nasopharyngeal carcinoma
AJCC: American Joint Committee on Cancer
BF: Blood flow
ROC: Receiver operating characteristic curve
T1WI: T1 weighted images
T2WI: T2 weighted images
T2WI-FS: T2 weighted fat suppression imaging
TR: Repetition time
TE: Echo time
FOV: Field of view
ROI: Region of interest
OAR: Organs at risk
EBV-DNA: Epstein-Barr virus DNA
GTV: Gross tumor volume
AUC: Area under curve
MR: Magnetic resonance
UICC: International Union Against Cancer
